# Delayed ulnar neuropathy in a child: imaging of post-fracture malunion and surgical fixation

**DOI:** 10.11604/pamj.2025.52.60.47454

**Published:** 2025-10-03

**Authors:** Nikita Gangwani, Subrat Samal

**Affiliations:** 1 Department of Musculoskeletal Physiotherapy, Ravi Nair Physiotherapy College, Datta Meghe Institute of Higher Education and Research, Sawangi, Meghe, Maharashtra, Wardha, India

**Keywords:** Tardy ulnar nerve palsy, pediatric elbow fracture, post-traumatic neuropathy

## Image in medicine

Tardy ulnar nerve palsy is a delayed-onset neuropathy caused by chronic compression or stretching of the ulnar nerve, commonly at the elbow. It often results from malunion or bony deformities following fractures, especially of the humerus and lateral epicondyle. The term "tardy" signifies the delayed appearance of symptoms, which may manifest months or years after the initial trauma. In pediatric populations, supracondylar and lateral epicondyle fractures of the humerus, often due to falls, are frequent. Improper healing may lead to deformities such as cubitus valgus or heterotopic ossification, predisposing children to progressive ulnar nerve entrapment. This report presents a 4-year-old male who developed progressive weakness and clawing of the fourth and fifth fingers of his left hand over two months. Five months earlier, he had sustained a supracondylar and lateral epicondyle humerus fracture from a fall, which was managed conservatively. Over time, his parents observed difficulty in grasping objects and an abnormal hand posture. Neurological examination revealed weakness in ulnar-innervated muscles, reduced grip strength, and sensory deficits along the ulnar distribution. There was no history of re-injury, and the child had normal developmental milestones. Imaging was performed to assess underlying bony abnormalities and nerve entrapment. This case highlights the importance of long-term follow-up in pediatric fractures to recognize post-traumatic neuropathies early. Timely diagnosis and intervention are essential to prevent permanent motor deficits and deformity in growing children.

**Figure 1:**
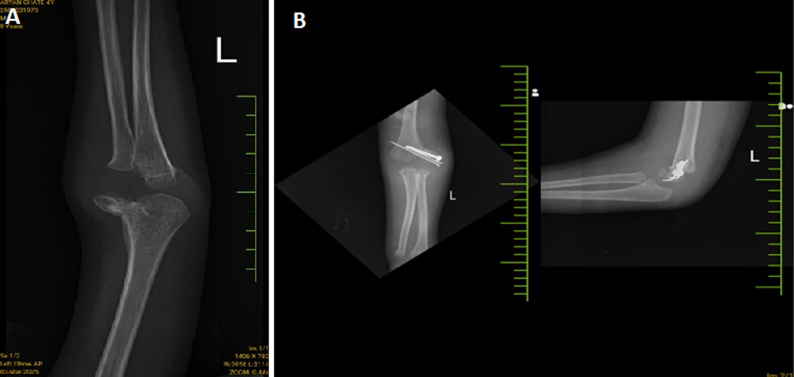
A) X-ray of Salter-Harris type II fracture of the radial head in a 4-year-old child´s left elbow, this type of fracture involves the growth plate and extends into the metaphysis, there appears to be some displacement of the radial head; B) anteroposterior and lateral X-ray views of the left elbow showing a malunited lateral epicondyle fracture surgically treated with osteotomy and K-wire fixation, additionally, a healed supracondylar humerus fracture with internal fixation is noted, heterotopic ossification is present anterior to the elbow joint, which may contribute to ulnar nerve compression

